# Inhibition of the Histone Methyltransferase EZH2 Enhances Protumor Monocyte Recruitment in Human Mesothelioma Spheroids

**DOI:** 10.3390/ijms22094391

**Published:** 2021-04-22

**Authors:** Silvia Mola, Giulia Pinton, Marco Erreni, Marco Corazzari, Marco De Andrea, Ambra A. Grolla, Veronica Martini, Laura Moro, Chiara Porta

**Affiliations:** 1 Department of Pharmaceutical Sciences, Università del Piemonte Orientale “Amedeo Avogadro”, 28100 Novara, Italy; silvia.mola@uniupo.it (S.M.); giulia.pinton@uniupo.it (G.P.); ambra.grolla@uniupo.it (A.A.G.); laura.moro@uniupo.it (L.M.); 2 Center for Translational Research on Autoimmune & Allergic Diseases (CAAD), Università del Piemonte Orientale “Amedeo Avogadro”, 28100 Novara, Italy; marco.corazzari@med.uniupo.it (M.C.); marco.deandrea@unito.it (M.D.A.); veronica.martini@uniupo.it (V.M.); 3 Unit of Advanced Optical Microscopy, IRCCS Humanitas Research Hospital, 20089 Rozzano, Italy; marco.erreni@humanitasresearch.it; 4 Department of Health Sciences, University of Piemonte Orientale “A. Avogadro”, 28100 Novara, Italy; 5 Interdisciplinary Research Center of Autoimmune Diseases (IRCAD), University of Piemonte Orientale, 28100 Novara, Italy; 6 Department of Public Health and Pediatric Sciences, Medical School, University of Turin, 10126 Turin, Italy; 7 Department of Translational Medicine (DIMET), University of Piemonte Orientale “A. Avogadro”, 28100 Novara, Italy

**Keywords:** malignant pleural mesothelioma, tumor-associated macrophages, monocytes, EZH2, spheroids, epigenetic reprogramming, tazemetostat, EPZ-6438, tumor microenvironment

## Abstract

Malignant pleural mesothelioma (MPM) is a highly aggressive cancer with a long latency period and dismal prognosis. Recently, tazemetostat (EPZ-6438), an inhibitor of the histone methyltransferase EZH2, has entered clinical trials due to the antiproliferative effects reported on MPM cells. However, the direct and indirect effects of epigenetic reprogramming on the tumor microenvironment are hitherto unexplored. To investigate the impact of tumor-associated macrophages (TAMs) on MPM cell responsiveness to tazemetostat, we developed a three-dimensional MPM spheroid model that recapitulates in vitro, both monocytes’ recruitment in tumors and their functional differentiation toward a TAM-like phenotype (Mo-TAMs). Along with an increased expression of genes for monocyte chemoattractants, inhibitory immune checkpoints, immunosuppressive and M2-like molecules, Mo-TAMs promote tumor cell proliferation and spreading. Prolonged treatment of MPM spheroids with tazemetostat enhances both the recruitment of Mo-TAMs and the expression of their protumor phenotype. Therefore, Mo-TAMs profoundly suppress the antiproliferative effects due to EZH2 inhibition in MPM cells. Overall, our findings indicate that TAMs are a driving force for MPM growth, progression, and resistance to tazemetostat; therefore, strategies of TAM depletion might be evaluated to improve the therapeutic efficacy of pharmacological inhibition of EZH2.

## 1. Introduction

Malignant pleural mesothelioma (MPM) is a rare and aggressive tumor that originates from mesothelial cells lining the pleural cavity. MPM has a strong causal link with exposure to airborne asbestos fibers and a long latency period of outcome; therefore, the incidence of MPM is expected to rise in the upcoming years, even though many developed countries have banned the use of asbestos in the late nineteenth century [1,2,3]. Based on cell morphology, MPM is classified into three major histologic subtypes with differences in prevalence and prognosis [4]. Epithelioid MPM is the most common and favorable subtype, although patients’ median survival is only 13 months after diagnosis [5]. The sarcomatoid is the rarest and most aggressive subtype, does not benefit from surgery, and has a median survival of 4 months, despite chemotherapy [6]. The biphasic is characterized by a mixed population of square-shaped (epithelioid) with elongated and spindle-shaped (sarcomatoid) cells; it accounts for 10–15% of MPM cases and has an intermediate prognosis that lies between the other two subtypes (median survival of 8 months) and might be linked with the proportion of the sarcomatoid component [4]. 

The long latency of tumor development, along with the nonspecific nature of the clinical symptoms, (e.g., pleural effusion, dyspnea, and chest pain) hinder the diagnosis; therefore, MPM is often detected at an advanced stage in elderly patients, further lowering the chance of a beneficial treatment [3,7]. MPM cells are highly resistant to conventional chemotherapy or radiotherapy, and surgery is rarely an option due to its complexity (extrapleural pneumonectomy, pleurectomy, or decortication) and the health status of the patients [8]. Despite the poor therapeutic benefits, chemotherapy based on pemetrexed and cisplatin has remained the cornerstone of its treatment since 2003; no second-line treatments have been approved yet [9]. Thus, the identification of new therapeutic strategies is an urgent medical need. 

Enhancer of zeste homolog 2 (EZH2) is the catalytic subunit of polycomb repressive complex 2 (PRC2) that plays a key role in the trimethylation of lysine 27 on histone H3 (H3K27me3), leading to chromatin compaction and gene repression [10]. In addition to being a crucial regulator of cell lineage determination [11], EZH2 is involved in several biological activities (e.g., cell cycle progression, autophagy, apoptosis, senescence, and repair of damaged DNA) and acts as a crucial oncogenic driver in many different malignancies [12]. EZH2 is frequently overexpressed in MPM, in which it represents both a useful biomarker for distinguishing MPM from reactive mesothelial hyperplasia [13,14] and a promising prognostic and therapeutic target [15]. Indeed, different studies have shown that high expression of EZH2 is associated with diminished patient survival [15,16], and its ablation or pharmacological inhibition significantly reduced proliferation, migration, clonogenicity, and tumorigenicity of MPM cells [16,17]. Hyperactivation of EZH2 due to gain of function mutations or overexpression has also been reported in different types of cancer, leading to the development of different EZH2 inhibitors [12]. Among these, tazemetostat/EPZ-6438 (Epizyme, Inc., Cambridge, MA, USA) has been recently approved by FDA for patients with advanced epithelioid sarcoma [18] and has also entered into clinical trials for diffuse large B-cell lymphoma [19], follicular lymphoma [20], and relapsed or refractory MPM with BRCA1 associated protein 1 (BAP1) inactivation [21,22]. 

Beyond the direct antiproliferative effect on tumor cells, EPZ-6438-driven epigenetic reprogramming can both directly and indirectly affect the tumor microenvironment (TME). Accumulating evidence highlights the importance of EZH2 in promoting cancer cell immune escape in different types of malignancies [23,24] and in shaping different tumor-infiltrating immune cells, overall supporting both immunosuppressive activities and antitumor immunity [25]. Thus far, the impact of EZH2 inhibition on human tumor-associated macrophages (TAMs) accumulation and functional activation is still largely unexplored. 

Macrophages play a key role in both MPM development and progression. In the attempt to clear asbestos fibers, “frustrated phagocytosis” triggers lung macrophages to release copious amounts of reactive oxygen species (ROS) and inflammatory cytokines, leading to increased recruitment and activation of immune cells and consequent generation of a chronic inflammatory state, promoting malignant transformation [26]. Macrophages are highly plastic cells; in response to local microenvironmental changes associated with tumor development and growth, tumor-associated macrophages (TAMs) can be reprogramed from powerful inflammatory and effector cells (M1-polarized activation) into immunosuppressive M2-like cells, supporting immune escape [27,28,29]. Accordingly, in MPM patients, the number of stromal macrophages positively correlates with Tregs [30] and the amount of CD163^+^ TAMs negatively correlated with overall survival [27,28]. Ablation of macrophages in mice also delayed tumor growth in different MPM models [27,31]. 

In addition to being central orchestrators of immunosuppressive TME [32], TAMs play a crucial role in dictating the efficacy of different therapies. Here, we developed a three-dimensional (3D) spheroid model consisting of human biphasic MSTO-211H cells and primary human monocytes to evaluate the effects of EZH2-dependent epigenetic reprogramming on TAM accumulation and activation, hence the impact of TAMs on MPM cell responsiveness to EPZ-6438.

## 2. Results

### 2.1. MSTO-211H Cells Form Multicellular Spheroids (MCSs) That Are Sensitive to EZH2 Inhibition and Capable of Recruiting Human Monocytes 

Multicellular spheroid (MCS) is increasingly appreciated as a useful in vitro model to evaluate drug response [33,34,35,36]. Based on this evidence, we decided to investigate the effects of TAMs on human biphasic MPM cell (MSTO-211H cell line) responsiveness to EPZ-6438 through a 3D MCS model. 

At first, we tested the effect of EPZ-6438 on MCS growth. We generated mCherry-expressing MSTO-211H and cultured these MPM cells in low adherence conditions, according to a well-established protocol [37]. 

**Figure 1:**
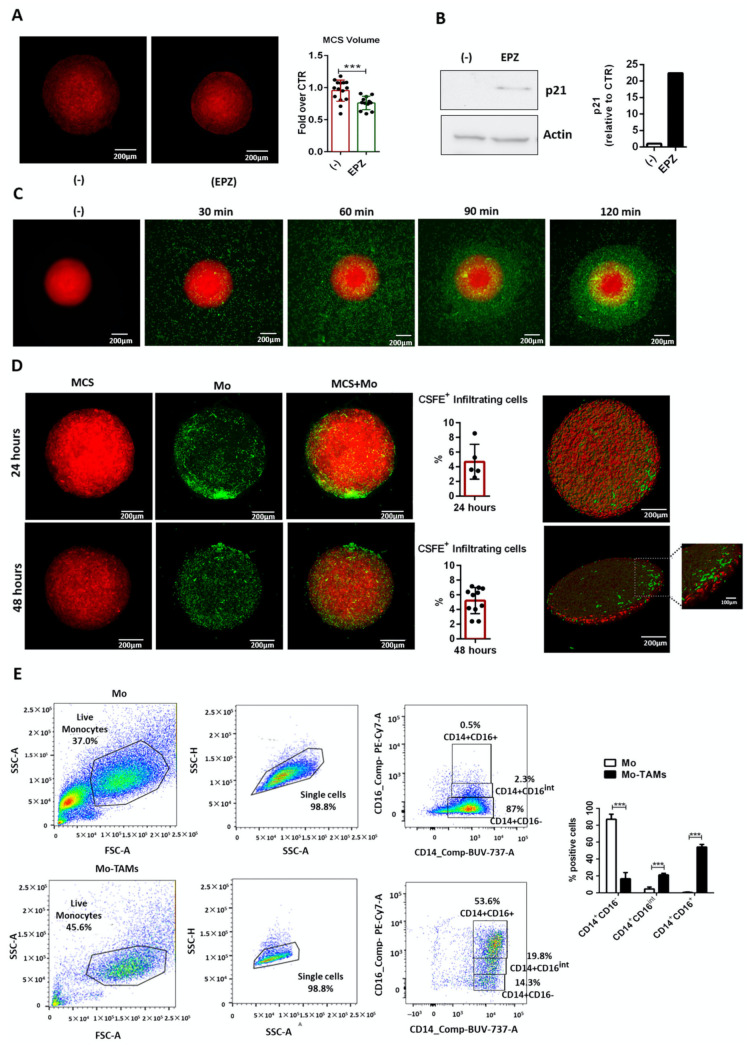
Human monocytes infiltrate MPM MCS (**A**) Volume of mCherry-MSTO-211H MCS treated with EPZ-6438 (EPZ, 10 μM) or vehicle (-) for 96 h was evaluated by confocal microscopy and calculated by ImageJ-win64 software. The results are shown as fold over and vehicle treated MCS (-). Data are shown as mean ± SD *** *p* < 0.001 by two-tailed *t*-test, (-) N = 11; EPZ N = 10. Representative images are shown. Scale bars: 200 μm; (**B**) Western blot analysis of p21 expression of MSTO-211H MCS, treated with 10 μM EPZ-6438 (EPZ) or vehicle (-) for 96 h. Total cell lysates were resolved by 10% SDS-PAGE. β-Actin was used as loading control; (**C**) migration of CSFE-labeled monocytes toward mCherry-MCS was monitored by time-lapse microscopy using Leica THUNDER Imager 3D Live Cell microscope equipped with an environmental chamber maintained at 37 °C, 5% CO_2_ humidified atmosphere. Representative frames at indicated times are shown. Scale bars: 200 μm; (**D**) mCherry-MCSs were cultured for 72 and 48 h, then cocultured with CSFE-labeled monocytes for an additional 24 or 48 h, respectively. MCSs were analyzed by confocal microscopy and infiltrating monocytes (CSFE^+^ cells) were quantified using Fiji-ImageJ (NIH). Representative confocal images after 48 h of coculture are shown. The 3D rendering was performed using Imaris (Bitplane) software. Scale bars: 200 μm or 100 μm. Data are shown as mean ± SD 24 h N = 5; 48 h N = 11; (**E**) FACS analysis of monocytes (CD14^+^ cells) cultured alone (Mo) or cocultured with MCS (Mo-TAMs) for 48 h to check their macrophage differentiation (CD14^+^CD16^+^ cells). Representative dot plot of Mo (upper panel) and Mo-TAMs (lower panel) are shown. Data are shown as mean ± SEM *** *p* < 0.001 by two-tailed *t*-test N = three different donors.

After 24 h, MSTO-211H cells assemble a 3D MCS that can be kept in culture for 4 days [37]. Therefore, mCherry-MCS were treated with EPZ-6438 or vehicle (-) [38] for 96 h and analyzed by confocal microscopy. Compared to the control (-), we observed that EPZ-6438 triggered a significant reduction in MCS volume (Figure 1A). In addition, Western blot analysis showed a consistent upregulation of the cell cycle inhibitor p21 in EPZ-6438-treated MCS (Figure 1B). These results confirm the antitumor activity of EZH2 inhibition in our MCS model. In order to gain more insight regarding the construction of an in vitro tumor model that better recapitulates TME, we evaluated whether human peripheral blood monocytes might be recruited into MCS. 

After 72 h of culture, carboxyfluorescein succinimidyl ester (CSFE)-labeled monocytes were added to mCherry-MCS and monitored by time-lapse imaging for 2.5 h. We observed that monocytes quickly migrated toward MCS, as demonstrated by their close localization around it as early as 2 h after coculture (Figure 1C). Confocal microscopy analysis demonstrated that monocytes were able to infiltrate the MCS after 24 h and were maintained in coculture for up to 48 h of (Figure 1D). 

Moreover, to test whether MCSs were able to induce macrophage differentiation, after 48 h of coculture, MCSs were disaggregated, and monocytes (CD14^+^ cells) were evaluated for the expression of the macrophage differentiation marker CD16 by FACS analysis. As expected, control monocytes were mainly CD14^+^CD16^−^ (86.9 ± 3.5%), whereas upon coculture, most of the CD14^+^ cells underwent macrophage differentiation as demonstrated by the large proportion of CD14^+^CD16^Int^ (21.1 ± 1.1%) and CD14^+^CD16^+^cells (54 ± 1.8%) (Figure 1E). 

Collectively, these results indicate that MCSs can both recruit human monocytes and promote their macrophage differentiation.

### 2.2. MCS-Infiltrating Monocytes Acquire a Functional TAM-Like Phenotype Supporting Tumor Growth

Different tumor cell lines produce soluble molecules that can skew the differentiation of human monocyte-derived macrophages (M-DM) toward a TAM-like phenotype [38]. 

**Figure 2:**
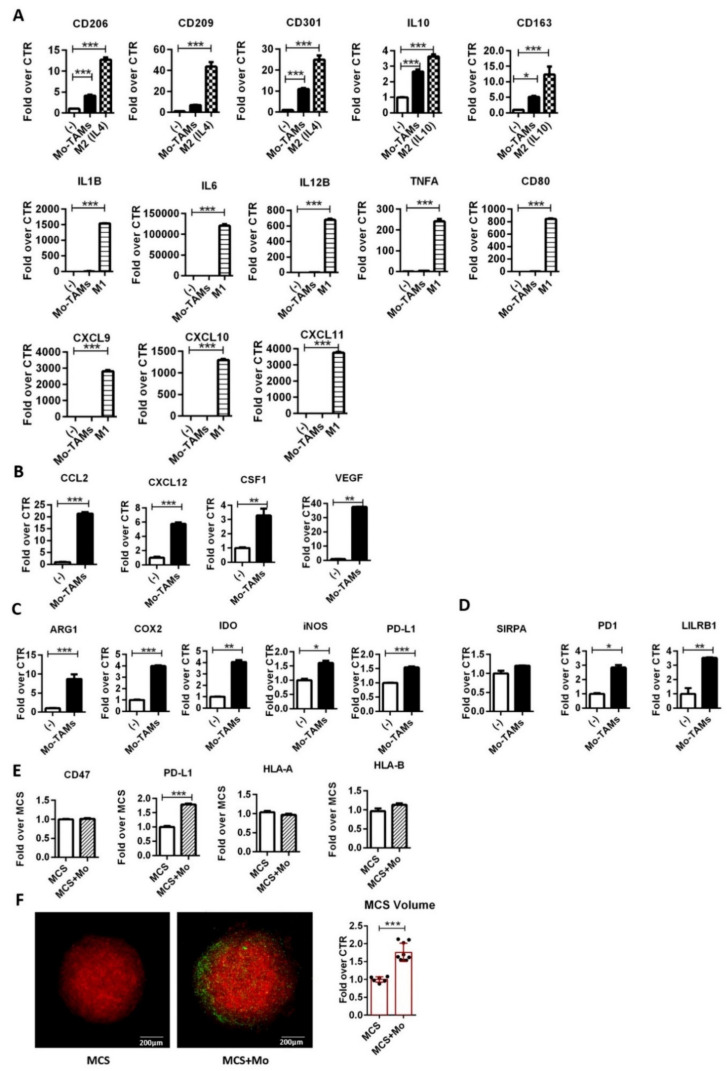
MCS-infiltrating monocytes acquire a functional TAM-like phenotype. (**A**–**D**) Monocytes-derived TAMs (Mo-TAMs) were isolated from MCS by magnetic cell sorting and analyzed by RT-PCR for the expression of genes encoding for selected (**A**) M1 (IL1, IL6, IL12B, TNFA, CD80, CXCL9, CXCL10, CXCL11), M2(IL4) (CD206, CD209, CD301), and M2(IL10) (IL10 and CD163) markers; (**B**) TAM-recruiting chemokines (CCL2 and CXCL12) and growth factors (CSF1 and VEGF); (**C**) immunosuppressive molecules (COX2, IDO, NOS2, ARG1, PD-L1); (**D**) immune checkpoint receptors (SIRPA, PD1, and LILRB1). M1-polarized monocytes by a 4 h treatment with LPS (100 ng/mL) and M2-skewed monocytes by an 18 h treatment with IL-4 (20 ng/mL) or IL-10 (20 ng/mL) were used as positive controls for M1 and M2 gene expression, respectively. β-Actin was used as housekeeping gene. Normalized RT-PCR results are shown as fold increase over untreated monocytes (-). Data are shown as mean ± SD and are representative of one out of three independent experiments with similar results. * *p* < 0.05, ** *p* < 0.01 and *** *p* < 0.001 by two-tailed one-way ANOVA or unpaired *t*-test, N = 3; (**E**) MSTO-211H cells isolated from MCS cocultured with monocytes were analyzed by RT-PCR for the expression of ligands for inhibitory phagocytosis receptors (CD47, PD-L1, HLA-A and HLA-B). β-Actin was used as housekeeping gene. Normalized RT-PCR results are shown as fold increase over MCS alone (MCS). Data are shown as mean ± SD and are representative of one out of three independent experiments with similar results. *** *p* < 0.001 by two-tailed unpaired *t*-test, N = 3; and (**F**) volume of mCherry-MCS culture without (MCS) or with CSFE-labelled monocytes (MCS + Mo) was evaluated by confocal microscopy and calculated by Fiji-ImageJ (NIH)-win64 software. The results are shown as fold-over MCS. Data are shown as mean ± SD *** *p* < 0.001 by two-tailed *t*-test. MCS N = 7 and MCS + Mo N = 9. Representative images are shown. Scale bars: 200 μm.

Therefore, we evaluated whether MCS could trigger the functional differentiation of infiltrating monocytes. After 48 h of coculture, MCSs were disaggregated and CD14^+^ cells (monocyte-derived TAMs) were magnetically sorted and analyzed for the expression of selected M1 and M2 genes by RT-PCR. Untreated, LPS (M1)-polarized, IL-4 (M2)-, or IL-10 (M2-like)- polarized monocytes were used as controls. The results showed significant induction of typical M2 genes (IL10, CD163, CD206, CD209, and CD301) in both monocyte-derived TAMs (Mo-TAMs) and IL-4 (M2)/IL-10 (M2-like) -treated monocytes, compared with the untreated ones. In contrast, all the genes encoding inflammatory mediators (e.g., TNFA, IL1B, IL6, IL12B, CD80, CXCL9, CXCL10, CXCL11) were highly induced in LPS-(M1) activated monocytes but were not significantly modulated in Mo-TAMs (Figure 2A). Overall, these results indicate that, similar to TAMs of MPM patients [32], in vitro Mo-TAMs have acquired an M2-skewed phenotype. Moreover, TAMs usually support their accumulation in solid tumors through the production of chemokines (e.g., CCL2 and CXCL12) and growth factors (e.g., CSF1 and VEGF). Accordingly, in our model, we observed a significant upregulation of these chemoattractants by Mo-TAMs, compared with control monocytes (Figure 2B). 

Next, we analyzed the expression of the major enzymes (COX2, IDO1, NOS2, ARG1) and immune checkpoints (CD-47/SIRPα [39], PD-L1/PD1 [40] and HLA class I/LILRB1 [41]), respectively, driving the immunosuppressive activity of tumor-infiltrating myeloid cells and hampering the ability of macrophages to eliminate cancer cells by phagocytosis (Figure 2C,D). In comparison with control monocytes, Mo-TAMs showed a consistently increased expression of COX2, IDO1, and ARG1 and a slight upregulation of NOS2 and PD-L1 (Figure 2C). These results along with the higher expression of IL10 and the low expression of the inflammatory cytokines indicate that Mo-TAMs have gained an immunosuppressive signature resembling human TAMs of MPM patients [32]. 

In addition, Mo-TAMs expressed SIRPA and showed a selective upregulation of PD1 and leukocyte immunoglobulin-like receptor LILRB1 (Figure 2D), two additional inhibitory receptors impairing macrophage-dependent clearance of cancer cells. In parallel, tumor cells isolated from MCS–monocytes coculture expressed the “do not eat me” signal CD47 as well as PD-L1 and HLA molecules (HLA-A and HLA-B), which are recognized ligands for PD-1 and LILRB1, respectively (Figure 2E). Therefore, these results suggest that tumor cells of MCSs are likely able to escape phagocytosis by Mo-TAMs. 

Overall, these results indicate that MCSs trigger infiltrating monocytes to express a protumor TAM-like phenotype. Therefore, we addressed the functional impact of Mo-TAMs on MCS growth. Confocal microscopy analysis showed a clear increase in mCherry^+^ MCS volume in coculture conditions, thus proving that Mo-TAMs support the growth of MCS (Figure 2F). 

Collectively, these results demonstrated that MCS-infiltrating monocytes functionally acquired a TAM-like phenotype endowed with tumor-promoting activities. Therefore, we have generated a reliable 3D model to investigate in vitro the effects of TAMs on biphasic MPM responsiveness to EPZ-6438.

### 2.3. EPZ-6438 Enhances Monocyte Recruitment in the MCS

**Figure 3:**
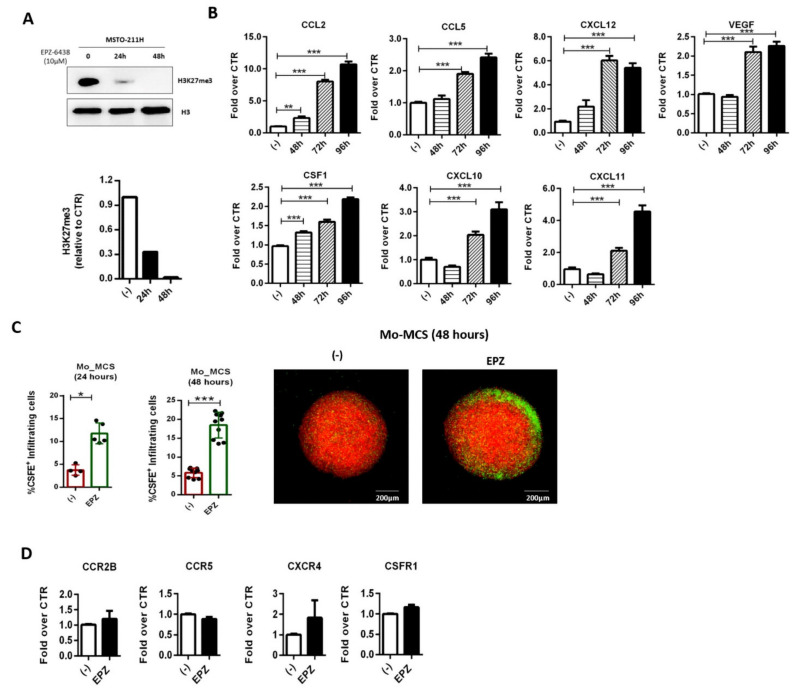
EZH2 inhibition enhances monocyte recruitment in MCS. (**A**) MSTO-211H cells were treated with EPZ-6438 (10 μM) or DMSO (-) for 24 and 48 h, then core histone proteins were extracted and analyzed by Western blot for H3K27me3 levels. Histone H3 was used as loading control; (**B**) RT-PCR analysis of total RNA extracted from MCS treated or not (-) with EPZ-6438 (10 μM) for 48, 72, and 96 h. Selected monocyte chemoattractants (CCL2, CCL5, CXCL12, CSF1, VEGF) were evaluated. β-Actin was used as housekeeping gene. Normalized qPCR results are shown as fold increase over control. Data are shown as mean ± SEM of three different experiments. *** *p* < 0.001 by two-tailed *t*-test, N = 3; (**C**) according to their time of life (about 96 h) MCSs were treated with EPZ-6438 (10 μM) for 72 or 48 h and subsequently cocultured with CSFE-labeled monocytes for 24 or 48 h, respectively. Infiltrating monocytes were quantified using Fiji-ImageJ (NIH)-win64 software. Representative images are shown. Scale bars: 200 μm. Data are shown as mean ± SD * *p* < 0.05 and *** *p* < 0.001 by two-tailed unpaired *t*-test; 24 h (-) N = 4, EPZ N = 5; 48 h (-) N = 7, EPZ N = 12; (**D**) monocytes were treated with EPZ-6438 (10 μM) for 48 h and analyzed for the expression of chemokine/growth factor-receptors (CCR2B, CCR5, CXCR4, and CSFR1) by qPCR. β-Actin was used as housekeeping gene. Normalized qPCR results are shown as fold increase over monocytes treated with DMSO only (-).

Cancer cell epigenetic reprogramming results in the expression of new transcriptional programs that impact antitumor immunity through different mechanisms [42]. In different cancer cell types, EZH2 inhibition increases Th1-recruiting chemokines expression, thereby shaping the composition of the tumor immune infiltrate [23,24]. Therefore, we evaluated whether EZH2 inhibition in MSTO-211H spheroids could modulate the expression of different immune cell chemoattractants, including chemokines (CCL2, CCL5, and CXCL12) and growth factors (CSF1 and VEGF) associated with monocyte recruitment and survival. Western blot analysis showed that H3K27 trimethylation of MSTO-211H cells was fully suppressed after a 24 h treatment with EPZ-6438 (10 μM) (Figure 3A). Therefore, tumor MCSs were cultured in the presence of EPZ-6438 (10 μM) or vehicle (-) for 48, 72, and 96 h, then RNA was extracted and analyzed by RT-PCR. Except for CXCL9, CCL17, and CCL22 transcripts, which were not detectable neither in basal nor in EPZ-6438-treated conditions, all the other genes encoding immune cell chemoattractants were expressed by MCS and maintained unaltered for the entire experimental period, in vehicle-treated conditions. In contrast, EPZ-6438 treatment enhanced CXCL10 and CXCL11 expression at later time points (Figure 3B). These results clearly indicate that similar to other tumor cells, EZH2 inhibition in MPM cells might enhance the recruitment of Th1 (CXCL10, CXCL11) but not of Th2 (CCL17, CCL22) cells. Noteworthy, we also observed a growing number of transcripts for the monocyte-recruiting molecules after 48 h (CCL2 and CSF1) or 72 h (CCL5, CXCL12, and VEGF) of EPZ-6438 treatment (Figure 3B). These results originally suggest that prolonged treatment with EPZ-6438 might increase TAM accumulation. 

To evaluate this hypothesis, we analyzed the recruitment of CSFE-labeled monocytes into mCherry-MCS, by confocal microscopy. According to the kinetics of expression of the chemoattractants and MCS time of life (about 96 h), mCherry-MCSs were treated with EPZ-6438 for 72 or 48 h and cocultured with CSFE-labeled monocytes for the following 24 and 48 h, respectively. In both experimental conditions, confocal microscopy analysis showed a consistent and significant increase in the infiltration of monocytes in EPZ-6438 treated MCS (Figure 3C).

Besides the effects of EZH2 inhibition on MSTO-211H cells, EPZ-6438 could also alter the responsiveness of monocytes to the chemoattractants. To address this aspect, we treated monocytes with EPZ-6438 for 48 h and we analyzed the expression of chemokine/growth factor receptors by RT-PCR. The results showed a similar mRNA level of CCR2, CCR5, CXCR4, and CSFR1 in both DMSO and EPZ-6438 treated monocytes, ruling out a direct effect of EPZ-6438 on the ability of monocytes to sense chemoattractants (Figure 3D). 

Overall, these results demonstrated that prolonged treatment of MCS with EPZ-6438 enhanced the expression of monocyte-recruiting molecules in association with an increased accumulation of tumor-infiltrating monocytes. 

### 2.4. EZH2 Inhibition Enhances Mo-TAM Protumor Phenotype

Epigenetic modulators can also directly modulate tumor-infiltrating immune cells [43]. Inhibition of EZH2 has been reported to be linked with enhanced NK and T cell effector activities in certain cancers [25], as well as with an increased expansion of myeloid-derived suppressor cells (MDSCs) and consequent suppression of antitumor immunity [44].

Macrophages are extremely plastic cells; therefore, we evaluated whether the epigenetic reprogramming associated with EZH2 inhibition might alter the protumor phenotype of TAMs. Thus far, the effects of EPZ-6438 on human monocyte–macrophage polarized activation have been poorly investigated [45]. Hence, we started by evaluating whether EZH2 inhibition might alter monocyte–macrophage responsiveness to either M1 (LPS) or M2 (IL-4 or IL-10) polarizing stimuli. 

**Figure 4:**
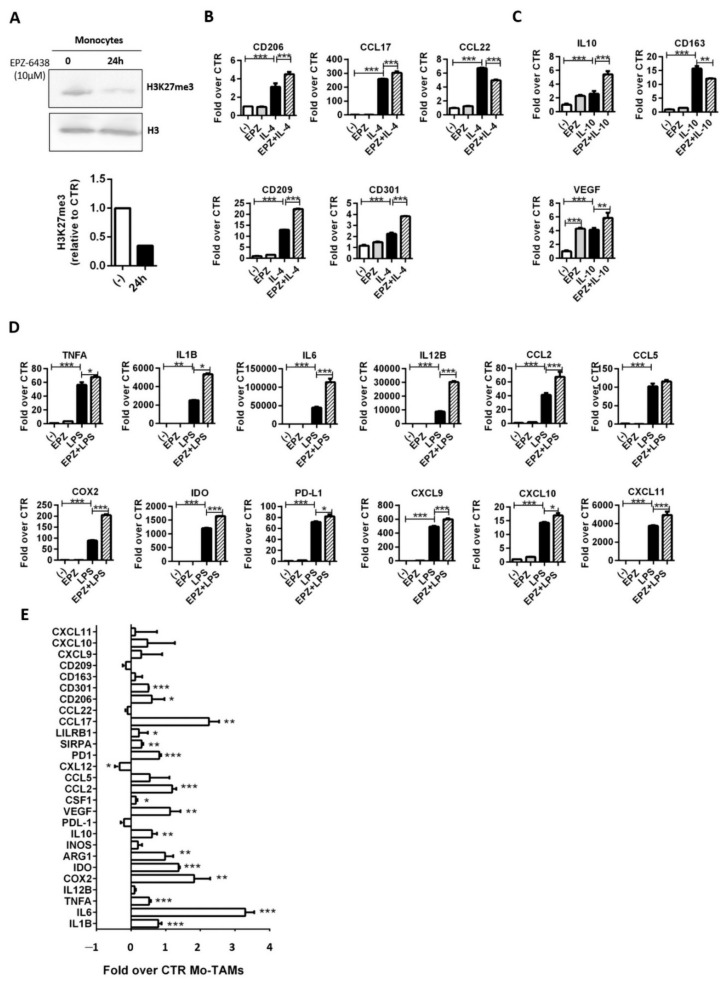
EZH2 inhibition affects human monocyte polarized activation. (**A**) Human monocytes were treated with 10 μM EPZ-6438 or DMSO (-) for 24 h, then core histone proteins were extracted and analyzed by Western blot for H3K27me3 levels. Histone H3 was used as loading control; (**B**,**C**) cells were pretreated with EPZ-6438 (10 μM) for 24 h and rechallenged with EPZ-6438 along with (**B**) IL-4 (20 ng/mL) or (**C**) IL-10 (20 ng/mL) for additional 18 h. Control cells were maintained in a medium supplemented with DMSO throughout the entire experimental period. An additional group of cells was treated with EPZ-6438 (10 μM) for 42 h and M2-polarized monocytes were maintained in a medium supplemented with DMSO for 24 h and then stimulated with (**B**) IL-4 (20 ng/mL) or (**C**) IL-10 (20 ng/mL) for the last 18 h. The expression of selected (**B**) IL-4 (CCL17, CCL22, CD206, CD209, and CD301) and (**C**) IL-10 (IL10, CD163, and VEGF) inducible genes was analyzed by RT-PCR. β-Actin was used as housekeeping gene. Normalized RT-PCR results are shown as fold increase over untreated monocytes (-). Data are shown as mean ± SD and are representative of one out of four independent experiments with similar results. ** *p* < 0.01 and *** *p* < 0.001 by two-tailed one-way ANOVA, N = 3; (**D**) monocytes were pretreated with EPZ-6438 (10 µM) for 20 h and then stimulated with LPS (100 ng/mL) for the following 4 h. Control cells were kept in culture for the entire experimental period. Additionally, a group of cells was treated with EPZ-6438 (10 µM) for 24 h and a group of cells was M1-polarized by LPS (100 ng/mL) stimulation during the last 4 h. Monocytes were analyzed to evaluate the expression of typical M1-inflammatory genes (TNFA, IL1B, IL6, IL12B, CCL2, and CCL5) by RT-PCR. β-Actin was used as housekeeping gene. Normalized RT-PCR results are shown as fold increase over untreated monocytes (-). Data are shown as mean ± SD and are representative of one out of four independent experiments with similar results. * *p* < 0.05, ** *p* < 0.01 and *** *p* < 0.001 by two-tailed one-way ANOVA; N = 3; (**E**) monocytes were coculture with MCS in presence of EPZ-6438 (10 µM) or DMSO only. After 48 h, Mo-TAMs were purified by magnetic separation and analyzed for the expression of selected genes including inflammatory cytokines (IL1, IL6, IL12B, TNFA), immunosuppressive molecules (IL-10, COX2, IDO, NOS2, ARG1, PD-L1), immune checkpoint receptors (SIRPA, PD1, and LILRB1), M2 markers (CD163, CD206, CD209, and CD301), monocyte (CCL2, CCL5, CXCL12, CSF1, VEGF), Th1 (CXCL9, CXCL10, CXCL11) and Th2 (CCL17, CCL22) chemoattractants. β-Actin was used as housekeeping gene. Normalized RT-PCR results are shown as fold increase over Mo-TAMs from DMSO treated MCS (-). Data are shown as mean ± SEM of three different experiments * *p* < 0.05, ** *p* < 0.01 and *** *p* < 0.001 by two-tailed *t*-test, N = 3.

Both primary human monocytes and M-DM, obtained by culturing peripheral blood monocytes with MCSF (40 ng/mL) for 6 days, were treated with EPZ-6438 (10 μM) for 24 h and analyzed by Western blot. In response to the EZH2 inhibitor, H3K27me3 dropped in both cell types (Figure 4A and Supplementary Figure S1A).

To analyze the effects of EZH2-dependent H3K27me3 on cell response to IL-4, monocytes, and M-DM obtained from different donors were treated or not with EPZ-6438 for 24 h and subsequently challenged with IL-4 (20 ng/mL) plus or not EPZ-6438 for an additional 18 h. Control cells were maintained in standard medium plus DMSO throughout the entire experimental period. An additional group of cells was cultured in the presence of EPZ-6438, for the entire experimental period, to evaluate the effects that are solely due to EZH2 inhibition.

We analyzed the expression of typical IL-4-induced genes by RT-PCR, and we found that both control and EPZ-6438-treated cells expressed comparable levels of the tested genes. In contrast, we observed a little upregulation of CD206, CCL17, CD209, and CD301 along with a slight downregulation of CCL22 in monocytes treated with EPZ-6438 plus IL-4, compared to those activated with IL-4 alone (Figure 4B). Similarly, in M-DM we confirmed that EPZ-6438 alone did not alter the expression of the selected IL-4 inducible genes, whereas EZH2 inhibition faintly modulated IL-4-induced gene expression (Figure S1B). 

We then analyzed the effects of EZH2 inhibition on monocyte response to IL-10, an immunosuppressive cytokine that is abundantly present in the MPM microenvironment [46]. 

In both monocytes and M-DM, we observed that EZH2 inhibition slightly increased IL-10-induced IL10 gene expression, whereas CD163 seemed to be EZH2 independent (Figure 4C and Figure S1C). Furthermore, in both cell types, we confirmed that EPZ-6438 was able to induce VEGF expression either alone or in combination with IL-10 (Figure 4C and Figure S1C). These results indicate that EZH2 modulates myeloid cell response to selected IL-10-induced genes.

Next, we explored whether the EZH2-dependent H3K27me3 selectively affects the expression of inflammatory (M1) genes. Human monocytes and M-DM were therefore cultured in presence of EPZ-438 (10 μM) for 24 h and activated with LPS (100 ng/mL) for the last 4 h. RT-PCR analysis confirms that EPZ-6438 alone does not affect gene expression, whereas pharmacological inhibition of EZH2 in both monocytes and M-DM sharply heightens the expression of selected inflammatory genes in response to LPS stimulation. Noteworthy, along with the antitumor cytokine IL12B, the other EZH2-dependent genes include monocyte recruiting chemokine (CCL2), protumor cytokines (IL1B, IL6), and immunosuppressive molecules (PD-L1, IDO1, COX2) (Figure 4D and Figure S1D).

Finally, to figure out the effect of EZH2 inhibition on Mo-TAMs, monocytes were cocultured with MCS in the presence or absence of EPZ-6438 for 48 h. In comparison with controls (Mo-TAMs isolated from DMSO-treated MCS), Mo-TAMs isolated from MCS exposed to the EZH2 inhibitor showed a significant upregulation of many protumor genes, including inflammatory cytokines (IL1B and IL6), immunosuppressive cytokines (IL10), and enzymes (COX2, IDO, and ARG1), inhibitory phagocytosis checkpoints (LILRB1, SIRPA, PD1) and selective M2 receptors (CD301, CD206) (Figure 4E). Furthermore, in keeping with the effect of EZH2 inhibition in tumor cells (Figure 3B), Mo-TAMs from EPZ-6438-treated MCS showed a consistent enhanced expression of monocyte chemoattractants CCL2 and VEGF but not Th1-recruiting chemokines (CXCL9, CXCL10, CXCL11). Instead, we confirmed a strong induction of the Th2-recruiting chemokine CCL17 we have reported with monocytes (Figure 4B) and M-DM (Figure S1B). Albeit we also observed a light induction of TNFA, (Figure 4E), overall, the results indicate that EPZ-6438 treatment strengthens the protumor phenotype of Mo-TAMs. 

### 2.5. Mo-TAMs Impair the Efficacy of EPZ-6438 

Given that MCS treatment with EPZ-6438 enhances the recruitment of monocytes and their functional differentiation in protumor cells, we evaluated the impact of TAMs on MPM cell responsiveness to EPZ-6438. 

**Figure 5:**
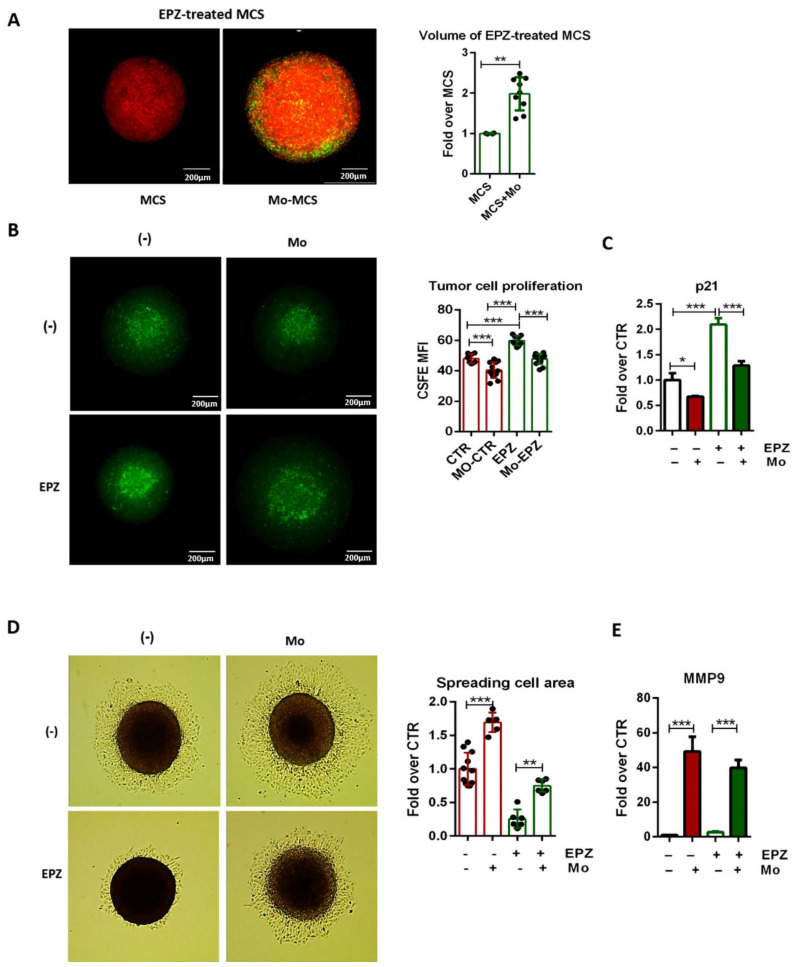
Mo-TAMs impair EPZ-6438 antitumoral effects. (**A**) mCherry-MCSs were treated with EPZ-6438 (10 μM) for 96 h and coculture or not with CSFE-labeled monocytes for the last 48 h. Volume of mCherry-MCS was evaluated by confocal microscopy and calculated by ImageJ-win64 software. Representative images are shown. Scale bars: 200 μm. Data are shown as mean ± SD ** *p* < 0.01 by two-tailed unpaired *t*-test MCSN = 4, MCS + Mo N = 9. (**B**) MCSs were generated with CSFE-labeled MSTO-211H cells and cultured for 96 h with 10 μM EPZ-6438 or DMSO (-). As indicated, monocytes were added and cocultured during the last 48 h. MCSs were analyzed by confocal microscopy then CSFE mean of fluorescence intensity (MFI) was quantified using Fiji-ImageJ (NIH)-win64 software. Representative images are shown. Scale bars: 200 μm. Data are shown as mean ± SD and are representative of one out of three independent experiments with similar results. *** *p* < 0.001 by two-tailed one-way ANOVA; (-) N = 8, EPZ N = 9, Mo N = 12, EPZ + Mo N = 13. (**C**) RT-PCR analysis of p21 expression by MSTO-211H cells derived from MCS cocultured with monocytes. β-Actin was used as housekeeping gene. Normalized RT-PCR results are shown as fold increase over MSTO-211H cells from DMSO treated MCS (-). Data are shown as mean ± SD and are representative of one out of three independent experiments with similar results. * *p* < 0.05 and *** *p* < 0.01 by two-tailed one-way ANOVA, N = 3. (**D**) MCSs were treated with 10 μM EPZ-6438 or DMSO for 48 h, then cocultured or not with monocytes for an additional 24 h and seeded in standard condition. After 24 h, spreading cells were analyzed by optical microscope and quantified by Fiji-ImageJ (NIH)-win64 software. Representative optical microscopic images are shown. Magnification: 10×. Representative data from one out of six independent experiments are shown. Data are shown as mean ± SD ** *p* < 0.01 and *** *p* < 0.001 by two-tailed one-way ANOVA; (**E**) RT-PCR analysis of MMP9 expression by MSTO-211H cells derived from MCS cocultured with monocytes. β-Actin was used as housekeeping gene. Normalized RT-PCR results are shown as fold increase over MSTO-211H cells from DMSO-treated MCS (-). Data are shown as mean ± SEM *** *p* < 0.001 by two-tailed *t*-test, N = 3.

We analyzed the volume of mCherry-MCS treated with the EZH2 inhibitor in comparison with those pretreated with EPZ-6438 for 48 h and subsequently cocultured with CSFE-labeled monocytes for an additional 48 h. Confocal microscopy analysis showed an approximately twofold increase of mCherry-MCS volume in coculture conditions (Figure 5A). These results indicate that Mo-TAMs impair the antiproliferative activity of EPZ-6438. To evaluate to what extent Mo-TAMs affect MSTO-211H cell responsiveness to EPZ-6438, we labeled MSTO-211H cells with CSFE and tracked their proliferation in the MCS. Confocal microscopy analysis confirmed that EPZ-6438 treatment reduced tumor cell proliferation, as evidenced by the decrease of CSFE intensity (Figure 5B). In contrast, Mo-TAMs triggered a similar tumor cell proliferation rate in both DMSO and EPZ-6438-treated MCS, indicating that their tumor-promoting effects had abolished the antiproliferative activity of EZH2 inhibition. To corroborate these observations, we evaluated the expression of the cell cycle inhibitor p21 in tumor cells by RT-PCR, and we confirmed that Mo-TAMs were able to fully revert the p21 induction triggered by EZH2 inhibition (Figure 5C). These results definitely validate the ability of Mo-TAMs to impair the antitumor activity of EPZ-6438. 

Strikingly, we noted that although MCSs were extremely resistant to both mechanic and enzymatic disaggregation, the presence of Mo-TAMs lowered spheroid firmness, allowing their disassembling into a viable single-cell suspension. This observation prompted us to investigate whether Mo-TAMs might affect the migratory behavior of MSTO-211H cells under both untreated and EPZ-6438 treated conditions. MCSs were pretreated or not with EPZ-6438 for 48 h, cocultured with monocytes for an additional 24 h, and subsequently seeded in standard conditions. After 24 h, we evaluated MSTO-211H cells grown around the MCS and found that Mo-TAMs greatly promoted tumor cell spreading under both DMSO and EPZ-6438 treated conditions (Figure 5D). Since both proliferative and migratory abilities likely contribute to determining the extent of tumor cells around the MCS, to investigate the effects of Mo-TAMs on tumor cell spreading behavior, we isolated MSTO cells from the MCS before seeding and analyzed the expression of selected genes associated with extracellular matrix (ECM) remodeling and epithelial-mesenchymal transition (EMT). Noteworthy, we found a dramatic upregulation of MMP9 in MSTO cells from MCS–monocytes coculture (Figure 5E), suggesting that Mo-TAMs might enhance the invasive ability of tumor cells by favoring the expression of the ECM degrading enzyme. The transcript levels of E-CADHERIN, N-CADHERIN, VIMENTIN, SNAIL, and MMP2 (Figure S2) were not significantly modulated, indicating the Mo-TAMs are unable to strengthen the mesenchymal phenotype of MSTO-211H cells; however, future studies with epitheliod MCS are warranted to evaluate the effect of Mo-TAMs on EMT better. Overall, these findings indicate that Mo-TAMs play a key tumor-promoting role in the biphasic MCS model, hampering EPZ-6438 efficacy and favoring MSTO-211H spreading capacity. 

### 2.6. MSTO-211H MCSs Neutralize the Cytotoxic Activity of M1-Polarized Monocytes

Immunotherapeutic strategies aimed at reprogramming TAMs into M1 effectors have been found effective in several preclinical models of cancers and are under evaluation in clinical studies for different types of human cancers [47,48]. Despite the fact that MPM cells are highly resistant to several chemotherapeutics, recent evidence indicates that LPS-activated RAW264.7 macrophages exert direct cytotoxic activity against MPM cells cocultured in standard 2D conditions [40]. 

Based on these premises, we started to evaluate the sensitivity of different types of human MPM cell monolayers to human M1-polarized monocytes.

**Figure 6:**
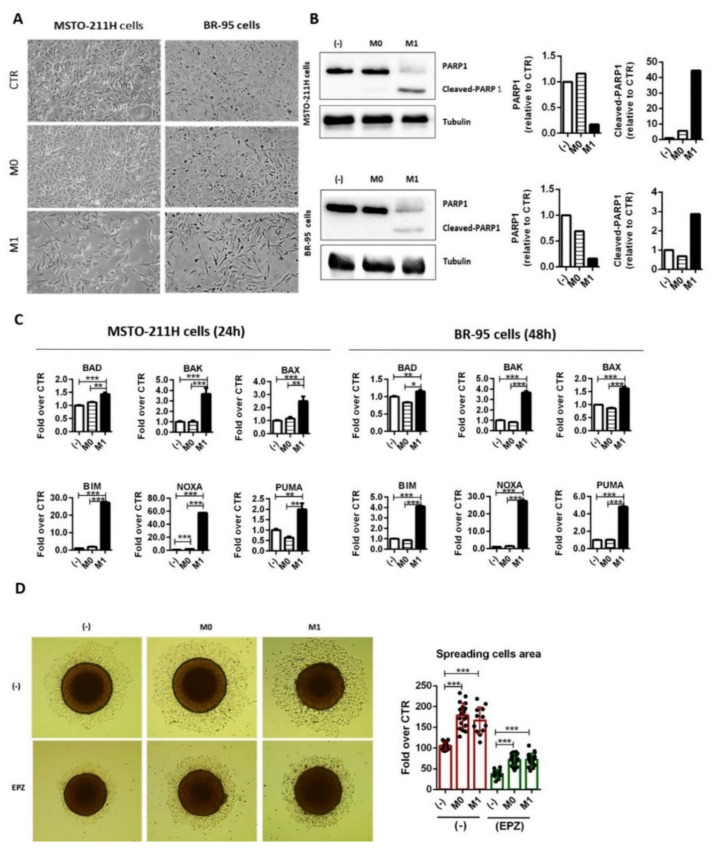
M1-polarized monocytes impair the survival of MPM cells grown as monolayer but not as MCS. (**A**,**B**) MSTO-211H and BR-95 cells were, respectively, cultured for 48 h or 72 h with 50% of control medium (ctr), M0, and M1-conditioned medium; (**A**) representative optical microscope images are shown. Magnification: 10×; (**B**) total proteins were extracted and resolved by 7% SDS-PAGE. PARP1 cleavages were analyzed by Western Blot. Tubulin was used as loading control; (**C**) total RNA was extracted from MSTO-211H cells and BR-95 cells treated with M0 or M1 monocytes-conditioned medium for 24 and 48 h, respectively. Proapoptotic gene expression (BAD, BAK, BAX, BIM, NOXA, PUMA) was evaluated by RT-PCR. β-Actin was used as housekeeping gene. Normalized RT-PCR results are shown as fold increase over untreated cells (-). Data are shown as mean ± SD and are representative of one out of three independent experiments with similar results. * *p* < 0.05, ** *p* < 0.01 and *** *p* < 0.001 by two-tailed unpaired *t*-test; (**D**) MCSs were treated with 10 μM EPZ-6438 or DMSO for 48 h, then cocultured or not with M0 or M1 monocytes for additional 24 h and seeded in standard condition. After 24 h, spreading cells were analyzed by optical microscope and quantified by ImageJ-win64 software. Representative optical microscopic images are shown. Magnification: 10×. Representative data from one out of six independent experiments are shown. Data are shown as mean ± SD *** *p* < 0.001 by two-tailed one-way ANOVA. (-) N = 15, M0 N = 19, M1 N = 12, EPZ N = 11, EPZ + M0 N = 19, EPZ + M1 N = 19.

Both biphasic MSTO-211H and epithelioid BR-95 MPM cells were treated with the medium (M1) collected from monocytes activated with INF-γ (20 ng/mL) and LPS (100 ng/mL) for 24 h. Similarly, tumor cells were treated with medium conditioned by naïve monocytes (M0) or with “control medium” obtained by incubating monocyte cell culture medium supplemented with LPS (100 ng/mL) and INF-γ (20 ng/mL) at 37 °C for 24 h. MSTO-211H and BR-95 cells treated with both control and M0-conditioned medium reached similar confluence after 48 h and 72 h of culture, respectively. In contrast, the treatment with an M1-conditioned medium led to a drastic reduction of attached cells, along with the presence of round floating cells (Figure 6A). These results indicate that M1 monocytes release soluble molecules capable of impairing the survival of both biphasic (MSTO-211H) and epithelioid (BR-95) cells. This hypothesis was confirmed by Western blot analysis of Poly ADP-ribose polymerase 1 (PARP-1) [49], a target of apoptotic caspases. Indeed, in both cell types, we observed that exposure to the M1-conditioned medium triggered a massive cleavage of PARP-1 (Figure 6B). In agreement, RT-PCR analysis showed a consistent increase in expression of genes encoding for the proapoptotic proteins BAX, BAK, NOXA, BIM, and PUMA in both MSTO-211H and BR-95 cell monolayers (Figure 6C). 

Overall, these results demonstrate that human M1 monocytes produce soluble factors capable of killing both biphasic and epithelioid MPM cell monolayers. Next, we moved to the more reliable MCS model in the attempt of evaluating whether the cytotoxic activity of M1 monocytes could synergize with the antiproliferative effect of EPZ-6438. Monocytes were M1 polarized by treatment with IFNγ (20 ng/mL) and LPS (100 ng/mL) for 4 h or maintained in culture untreated (M0). Next, untreated or EPZ-6438-treated MCS were cocultured with M0 or M1 monocytes for 24 h and subsequently, seeded in standard conditions for an additional 24 h. The results showed that both M1 and M0 monocytes similarly enhance tumor cell spreading and hamper the antitumor effect of EPZ-6438 (Figure 6D). These results suggest that MPM cells in the MCS were resistant to the cytotoxic activity of M1-polarized monocytes or were able to reeducate M1-monocytes in protumor Mo-TAMs. Therefore, to enhance the therapeutic effect of EPZ-6438, other strategies aimed at inhibiting TAM accumulation rather than reprogramming Mo-TAMs in M1 effectors might be considered in combination with EZH2 inhibition. 

## 3. Discussion

EPZ-6438 is an inhibitor of the histone methyltransferase EZH2 that has recently entered clinical trials for MPM patients due to its antiproliferative effects on tumor cells [21,22]; however, the impact of this epigenetic modulator on the TME and the effects of TME on MPM cell responsiveness to EZH2 inhibitors are still largely unexplored. 

Macrophages represent the major population of the MPM immune cell infiltrates and are the driving force for the construction of an immunosuppressive TME favorable for tumor progression [28,30]. TAMs can also deeply affect the efficacy of anticancer therapies [50]. Although epithelioid MPM is the most common subtype, the sarcomatoid cells are the most aggressive and resistant tumor cell types; therefore, we focused our studies on MSTO-211H cells, a spindle-shaped MPM cells that represent the sarcomatoid component of biphasic MPM. Here, we provide evidence that inhibition of EZH2 enhances the capacity of MPM to recruit monocytes and to drive their differentiation toward a TAM-like phenotype, which in turn impairs the antitumor effects of EPZ-6438. 

Despite studies involving research animals remain a crucial step forward in the development of new drugs, the growing awareness of the *Principles of Humane Experimental Technique*, the 3Rs, has triggered the development of new technologies and models to improve the capacity of addressing biological questions in vitro. In comparison to monolayer cell cultures, MCSs are 3D models that accurately mimic many features of solid tumors (e.g., gradients of nutrients, oxygen, and pH establish over time, ECM deposition, cell–cell and cell–ECM interactions, cellular layered assembling) leading to a gene expression signature and drug resistance mechanisms that closely resemble those of patient-derived tumors [33,36,51]. As a further improvement, we developed a 3D MPM spheroid model that recapitulates in vitro, both monocyte recruitment in tumors and their functional differentiation in TAMs. Indeed, similar to TAMs of MPM patients, MCS-infiltrating monocytes (Mo-TAMs) acquire a transcriptional profile characterized by a selective high expression of genes encoding M2-like receptors (CD163, CD206, CD301, CD209) and immunosuppressive molecules (IL-10, COX2, IDO1, ARG1, iNOS, PD-L1) [28,30]. Mo-TAMs also upregulated the expression of different chemokines (CCL2, CXCL12) and growth factors (CSF1, VEGF), which are crucial drivers of monocyte recruitment, survival, and M2-skewed activation. Noteworthy, CCL2, a key monocyte chemoattractant, is significantly elevated in both pleural effusion [52] and serum of MPM patients in advanced stages of the disease [40]. Moreover, CSF-1R is highly expressed in human TAMs [53], further corroborating their similarity to Mo-TAMs. According to MPM patients [39], we also observed that both SIRPα and the “do not eat me” signal CD47 were, respectively, expressed by Mo-TAMs and tumor cells. CD47-SIRPα is the best-characterized phagocytosis inhibitory axis, whose neutralization has been demonstrated effective across multiple preclinical models and early phase clinical trials [54]. However, additional immune checkpoints have emerged as key repressors of macrophage effector functions and promising targets for cancer immunotherapy [55]. Noteworthy, we observed that, in comparison with control monocytes, Mo-TAMs significantly upregulated the phagocytosis checkpoints PD1, whose expression in RAW264.7 macrophages has been recently reported to be associated with their impaired ability to uptake mouse MPM cells [40]. In addition, we found that Mo-TAMs heightened the expression of LILRB1, a hitherto uncharacterized inhibitory receptor in the context of MPM disease, that recognizes MHC class I complexes and impairs the ability of TAMs to clear different types of cancer cells both in vitro and in vivo [41]. Overall, these data support the notion that MCSs trigger the infiltrating monocytes to acquire a TAM-like phenotype endowed with tumor-promoting activities.

Accordingly, functional assays actually demonstrated that Mo-TAMs enhance MCS growth by increasing tumor cell proliferation, as indicated by the reduction of both CSFE intensity and p21 levels. Mo-TAMs also reduced MCS firmness, enhancing tumor cell spreading. These results suggest that Mo-TAMs modulate the remodeling of the extracellular matrix, creating a TME that is more permissive for tumor cell invasion. In support of this hypothesis, we observed that Mo-TAMs triggered a strong induction of MMP9 expression by MPM cells and clinical studies have reported that high expression of MMP9 correlates with a more aggressive MPM phenotype and decreased patients’ survival [56,57].

Definitively, we have generated a reliable 3D model that can be used to evaluate in vitro the impact of protumoral TAMs on MPM cell response to antiproliferative drugs. However, anticancer agents targeting epigenetic modulators, in addition to affecting tumor cells directly, can profoundly modulate the composition and the functional activation of tumor-infiltrating immune cells that consequently improve or impair drug efficacy [42,43].

Along with epigenetic silencing of tumor-suppressor genes [12], in different types of cancer cells, EZH2 upregulation is linked with the transcriptional repression of genes that confer immunogenicity (e.g., MHC I and II) and support the recruitment of effector T cells (e.g., CXCL9 and CXCL10) [24,58,59,60]. Therefore, EZH2 inhibition alone or in combination with immune checkpoints has been found to be associated with enhanced CD8 T cell infiltration and antitumor immunity [23,24,61,62]. In line with these findings, we have observed the upregulation of Th1 recruiting chemokines (CXCL10, CXCL11) in EPZ-6438-treated MCS, but we have also found a significantly increased expression of different chemokines (CCL2, CCL5, CXCL12) and growth factors (CSF-1 and VEGF) promoting recruitment, differentiation, and survival of monocytes in tumors. These transcriptional events actually correlate with an enhanced accumulation of monocytes in MCS that, due to their protumoral phenotype, curb the antiproliferative effects of EZH2 inhibition in cancer cells. These results suggest that in vivo treatment with tazemetostat might enhance TAM infiltrate in biphasic MPM. Whether this event could occur along with increased recruitment of antitumor T cells and the overall impact of immune cells on clinical response are important questions that warrant future studies. 

EZH2-dependent chromatin remodeling also directly shapes immune cell fate and functions [25]. However, understanding the overall impact of EZH2 inhibition on antitumor immunity requires consideration of its pleiotropic effects on different types of immune and tumor cells. EZH2 modulates gene expression programs that stabilize the functional phenotype of Treg [63,64,65] and limit the differentiation and functional activation of NK cells [66,67]. Therefore, its inhibition has been found to be associated with an enhanced antitumor immunity in certain cancers. Nevertheless, EZH2 expression supports the activity of tumor-specific effector T cells in ovarian cancer [68] and restrains the expansion of MDSCs in murine models of lung and colon cancer [44], indicating that EZH2 inhibitors can also impair antitumor immunity. In glioblastoma cells, EZH2 inhibition promotes the release of soluble factors capable of shifting human M-DM and murine microglial cells toward an M1 antitumor phenotype [69]. In contrast, our results indicate that Mo-TAMs isolated from EPZ-6438-treated MPM spheroids have an enhanced protumor phenotype, as demonstrated by the faint induction of M1 antitumor gene TNFA, along with a consistent upregulation of protumor cytokines (IL-1β, IL-6), immunosuppressive molecules (IL10, COX2, IDO, and ARG1) and M2-like products (CD301, CD206, CCL17). In keeping with the effect of EZH2 inhibition in tumor cells, Mo-TAMs from EPZ-6438-treated MCS also showed a significantly enhanced expression of CCL2 and VEGF, thus suggesting a potentiation of the circuits favoring TAM accumulation. In agreement with EZH2-dependent repression of PD1 in murine RAW264.7 macrophages [40], we confirmed that human Mo-TAMs from EPZ-6438-treated MCS express higher levels of multiple phagocytosis inhibitory receptors (PD1, LILRB1, and SIRPA), therefore indicating an additional impairment of macrophage effector functions. Overall, in the MPM spheroid model, pharmacological inhibition of EZH2 strengthens the protumoral phenotype of Mo-TAMs. Therefore, under EPZ-6438 treatment, both tumor cell proliferation and spreading ability are significantly rescued by Mo-TAMs. 

Mechanistically, the effect EPZ-6438 might be due to both a modified expression of polarizing signals by MPM cells and altered responsiveness of Mo-TAMs to the TME signals.

EZH2 inhibition in tumor cells of MCS increases the expression of CSF-1 that, beyond enhancing TAM accumulation, is a crucial TME signal skewing their differentiation toward an M2 phenotype [32,70]. Accordingly, in different cancer types, CSF-1R blockade has been found to be associated with TAM reprogramming in antitumor effectors [53,71,72].

In human macrophages, the effect of EZH2-driven chromatin remodeling on cell response to different polarizing stimuli is still poorly explored. In response to IFNγ stimulation, EZH2-driven H3K27me3 stably represses selected anti-inflammatory genes (MERTK, PPARG, and RANK) that become refractory to activation by the inducers [45]. Consistent with Mo-TAMs, we found that in both human monocytes and M-DM, the epigenetic reprogramming driven by EPZ-6438 selectively enhances the expression of genes that are induced by LPS (IL1β, IL6, IL12B, TNFA, CCL2, COX2, IDO), IL-4 (CD206, CD301, CCL17), and IL-10 (IL10, VEGF), thus indicating that EZH2 controls the transcription of a limited number genes that are activated by both M1 and M2 stimuli. Our results propose that the TME signals of MCS foster the expression of protumor genes, therefore limiting the antitumor activity of EZH2 inhibition in MPM cells. Certainly, these findings point out a dual activity of EPZ-6438 in MPM. Given that the beneficial antiproliferative effects of EZH2 inhibition in MPM cells are counteracted by the enhanced accumulation of protumoral Mo-TAMs, strategies of TAMs reprogramming in M1 antitumor effectors would ideally work in synergism with EZH2 inhibitors. Consistent with previous studies with murine RAW264.7 macrophages [40], we observed that M1-polarized monocytes release soluble factors capable of killing different MPM cell monolayers. However, in the 3D MCS model, tumor cells are highly resistant to the cytotoxic activity of M1 monocytes. The observation that naïve (M0) and M1 polarized monocytes exert comparable protumor effects, suggests that MCS fully reprogram M1 monocytes in protumoral Mo-TAMs. Although strategies capable to enhance TAM effector capacity efficiently would be advisable, inhibition of monocyte recruitment in MPM and TAM depletion represent alternative valuable approaches that could be exploited to improve biphasic/sarcomatoid MPM response to EPZ-6438. Different strategies to eliminate TAMs selectively have been significantly advanced toward the clinic [73,74,75] and have been preliminarily evaluated as single agents in MPM [76,77]; their potential combination with tazemetostat might hopefully open new therapeutic perspectives for the more aggressive MPM subtypes.

## 4. Materials and Methods

### 4.1. Isolation and Purification of Human Monocytes

Monocytes were isolated from buffy coats of healthy donors, using sequential Ficoll and Percoll density gradient centrifugations, as previously described [78].

For gene expression or Western blot analysis, cells were seeded in an incomplete RPMI medium and incubated at 37 °C and 5% of CO_2_. After 1 h, monocytes were selectively attached to the plate, and nonadherent cells were vigorously washed out with saline. Monocytes were cultured in RPMI 1640 supplemented with 1% L-glutamine and 1% penicillin–streptomycin and 10% of FBS and treated as follows.

For coculture with MPM spheroids, after Percoll gradient, CD14^+^ monocytes were purified by using Miltenyi Biotech CD14 MicroBeads kit, for positive magnetic separation, according to the manufacturer’s instructions.

### 4.2. In Vitro Generation of Monocytes-Derived Macrophages (M-DM)

CD14^+^ monocytes were seeded in complete RPMI at a concentration of 2 million cells/mL in low adherence plates and cultured in the presence of human M-CSF (40 ng/mL, Miltenyi) for 6 days. Macrophage differentiation was evaluated by flow cytometry. Cells were stained in 0.5% FBS, HBSS solution with anti-human CD14-FITC (clone M5E2, Bio-Legend) and anti-human CD16-PE (clone 3G8, Bio-Legend) and analyzed by an S3 Cell Sorter (Biorad). When more than 90% CD14^+^ cells coexpressed the macrophage differentiation marker CD16, M-DMs were used, as described in the following sections.

### 4.3. MPM Cells Lines 

The MSTO-211H cell line, which is derived from biphasic MPM and harbors a fibroblast-like morphology, was obtained from the Istituto Scientifico Tumori (IST) Cell-bank of Genoa, whereas the epithelioid BR-95 cell line was obtained from the Biobank of the Hospital of Alessandria. All cell lines were routinely tested for mycoplasma contamination by PCR. Cells were grown in RPMI 1640, supplemented with 1% L-glutamine and 1% penicillin–streptomycin and 10% of FBS, and placed in a 37 °C humidified incubator with 5% CO_2_.

### 4.4. Cell Treatments

To evaluate the effect of EZH2 inhibition on H3K27me3, MSTO-211H, monocytes, M-DMs were treated with EPZ-6438 (10 µM, Selleckchem, Houston, TX, USA) for the indicated time, then core histone proteins were extracted and analyzed by Western blot. Control cells were treated with the same amount of DMSO (Sigma-Aldrich, Saint Louis, MO, USA). Due to the low stability of EPZ-6438, for treatments longer than 24 h, cells were rechallenged with the same dose of drug or vehicle (DMSO) every 24 h.

To evaluate the effect of EPZ-6438 on M1-polarized activation, monocytes were treated with EPZ-6438 (10 µM) for 24 h and stimulated with LPS (100 ng/mL, Enzo Life Sciences, Farmingdale, NY, USA) during the last 4 h. Control cells were kept in culture for the entire experimental period. In addition, cells were treated with EPZ (10 μM) for 24 h or maintained untreated for 20 h and stimulated with LPS (100 ng/mL) for the last 4 h. 

To evaluate the effect of EPZ-6438 on M2-polarized activation, monocytes were pre-treated with EPZ-6438 (10µM) for 24 h and rechallenged with EPZ-6438 along with IL-4 (20 ng/mL, Miltenyi, Biotech, Bergisch Gladbach, Germany) or IL-10 (20 ng/mL, Miltenyi) for the following 18 h. Control cells were kept in culture for the entire experimental period. Additionally, cells were treated with EPZ-6438 (10µM) for 42 h, and cells were maintained untreated for 24 h and stimulated with IL-4 or IL-10 (20 ng/mL) for the last 18 h. 

### 4.5. Production of Monocytes Conditional Media and Treatment of MPM Cells

Monocytes isolated from buffy coat were cultured in complete RPMI 1640. To induce M1 polarized activation, cells were stimulated with h-IFNγ (20 ng/mL, Miltenyi) and LPS (100 ng/mL, Enzo Life Science). After 24 h, the medium was collected, centrifuged at 1400 rpm, RT, 10 min, filtered, and stored at −20 °C in aliquots until use. In the same manner, aliquots collected from untreated monocytes (M0) and control RPMI 1640 medium containing the polarizing stimuli and left in the incubator for 24 h were stored. 

MSTO-211H and BR-95 cells were plated in 100 mm dishes in complete RPMI 1640, until 60% of confluence, then culture medium was replaced with fresh medium containing 50% of control, M0, or M1 monocytes-conditioned medium. Cells were monitored for the following 48–72 h by optical microscope. To evaluate cell viability, cells were harvested, and proteins and total RNA were extracted and analyzed by Western Blot and RT-PCR, respectively.

### 4.6. Generation of m-Cherry MSTO-211H Cells

mCherry expressing MSTO-211H cells were generated by infecting MSTO-211H cells with a third-generation lentiviral vector that constitutively expressed the fluorescent reporter mCherry. HEK293T cells were transfected with pRRLsinPPTCherry_peo, pMDL, pREV, and pVSVG plasmids by using Lipofectamine 2000 (Invitrogen, Life Technologies Corporation, Eugene, OR, USA) according to the manufacturer’s instructions. After 48 h, the medium, containing the viral particles, was collected, filtered, and ultracentrifuged at 70,000 *g*, +4 °C, 2 h. The lentiviral pellet was resuspended in complete medium and directly added on semiconfluent MSTO-211H cells. Infected mCherry MSTO-211H were selected by cell sorting (S3 Cell Sorter, Bio-Rad Laboratories, Hercules, CA, USA), expanded, and maintained in culture in complete RPMI 1640, in a 37 °C humidified incubator with 5% CO_2_. 

### 4.7. MPM Multicellular Spheroids (MCSs)

MCSs were generated according to a published protocol [37] with minor modifications. Specifically, we coated the 96-well plates with a thin layer of 1% sterile agarose (Sigma) in water solution. Before use, the coated plates were sterilized by UV light for 20 min. In each well we seeded 10.000 MSTO-211H cells resuspended in 100 μL of RPMI 1640 medium, supplemented with 1% L-glutamine and 1% penicillin–streptomycin and 10% of FBS. After overnight culture in a humidified incubator, at 37 °C and 5% CO_2_, MSTO-211H cells aggregated to form an MCS/well. MCS were kept in culture for 96 h maximum, according to the experimental procedures described in the following section.

### 4.8. Coculture of MSTO-211 Spheroids and Monocytes

MCSs were pretreated for 48 h with EPZ-6438 (10 µM) or DMSO, and then 50,000 monocytes were added to each MCS and cocultured for an additional 48 h in the presence or absence of EPZ-6438. MCSs were mechanically disaggregated and both CD14^+^ (monocyte-derived TAMs) and CD14^−^ (tumor cells) cells were isolated by using Miltenyi Biotech CD14 MicroBeads kit, for positive magnetic separation, according to the manufacturer’s instructions. Both cell populations were analyzed for gene expression by RT-PCR. 

MCSs were kept untreated (DMSO challenged) or treated with EPZ-6438 (10 µM) for the entire experimental period.

### 4.9. Readherence of MSTO-211H Spheroids

Monocytes were left untreated (M0) or were M1-polarized by IFNγ (20 ng/mL) and LPS (100 ng/mL) treatment for 4 h. 

MCSs were pretreated for 48 h with EPZ-6438 (10 μM) or DMSO, and then 30,000 monocytes were added to each MCS and cocultured for an additional 24 h in the presence or absence of EPZ-6438. Next, MSTO-211H spheroids were plated in standard conditions and cultured for the last 24 h_._ To evaluate the proliferating cells around the spheroid, the images were captured using an optic microscope (Leica MB5000B), and the area of spreading cells was quantified by Fiji-ImageJ (NIH)-win64 software (National Institutes of Health, Bethesda, MD, USA). 

### 4.10. Total Protein Extraction

Cells were washed twice with cold saline solution and lysed by directly adding 1% NP-40 lysis buffer (1% NP-40, 150 mM NaCl, 50 mM Tris-HCl pH 8.5 10 mM EDTA, 10 mM NaF, 10 mM Na_4_P_2_O_7_, 0.4 mM Na_3_VO_4_) with freshly added protease inhibitors (10 μg/mL leupeptin, 4 μg/mL pepstatin and 0.1 Unit/mL aprotinin). Cell lysates were collected and centrifuged at 13,000 rpm, +4 °C, for 20 min. The pellets were discarded and the supernatants containing the proteins were collected and assayed for protein concentration with the Bradford method.

### 4.11. Acidic Extraction of Histone Core Proteins

Cells were collected and washed twice with cold saline. The pellet was then resuspended in 1× packed bed volume of ice-cold 5% perchloric acid lysis solution (5% PCA, 10 mM N-ethylmaleiamide, 10 mM NaF, 10 mM Na_4_P_2_O_7_, 0.4 mM Na_3_VO_4,_ Cocktail α-proteasi 1×), kept on ice for 10 min and centrifuged at 13,000 rpm, +4 °C, for 10 min. The extraction was repeated three times. The remaining pellet was resuspended in 2× packed bed volume of an ice-cold solution of HCl 0.4 N, 10 mM N-ethylmaleiamide, and protease inhibitors, centrifuged at 13,000 rpm, +4 °C, for 10 min. The extraction was repeated three times. Supernatants were collected and histone core proteins were precipitated with an ice-cold solution of 25% trichloroacetic acid (TCA) on ice for 30 min and collected by centrifugation at 1300 rpm, +4 °C, 20 min. The protein pellet was sequentially washed with 1 mL of ice-cold 100% acetone +0.006% HCl solution and 1 mL of ice-cold 100% acetone solution. 

The dry pellet was resuspended in Tris-HCl (50 mM) and quantified with the Bradford assay method.

### 4.12. Immunoblot

Proteins were separated by SDS-PAGE under reducing conditions, transferred onto nitrocellulose membrane, and incubated with H3K27me3 (ab6002, Abcam), H3, (ab1791, Abcam), PARP1, (sc8007, Santa Cruz, Dallas, TX, USA), p21 (C-19, Santa Cruz), α-Tubulin (Sigma-Aldrich, Saint Louis, MO, USA), β-Actin (A1978, Sigma-Aldrich) antibodies. After incubation with peroxidase-conjugated Goat-α-Rabbit (7074S, Cell Signaling Technology, Danvers, MA, USA), Goat-α-Mouse (7076S, Cell Signaling) antibodies, chemiluminescent ECL reagent (Biorad) were added, and the signals were detected at transilluminator Chemidoc (Biorad).

### 4.13. Gene Expression Analysis

Total RNA was extracted according to the manufacturer’s instructions of RNeasy Mini Kit (Qiagen, Hilden, NRW, Germany). 

RNA was quantified by using a Nanodrop spectrophotometer. RNA quality was assessed as 260/280 and 260/230 OD ratio >1.5, 1 μg of RNA was reverse transcribed by the Script Advanced cDNA Synthesis Kit (Bio-Rad Laboratories, Hercules, CA, USA). In addition, 20 ng of template was amplified using SYBER Green Master Mix (Bio-Rad) and detected by the CFX96 Real-Time System (Bio-Rad). All samples were run in triplicate. Expression data were normalized to β-Actin housekeeping mRNA and were analyzed by the 2^−(ΔCt)^ method. Results are expressed as fold upregulation with respect to the control cell population. The sequences of gene specific were designed by using https://www.ncbi.nlm.nih.gov/tools/primer-blast/ (accessed on 1 January 2018). The primers were synthesized by Sigma-Aldrich; the sequences are reported in Supplementary Table S1.

### 4.14. Fluorescence-Activated Cell Sorting (FACS) Analysis

A total of 1 × 10^6^ cells were stained with antigen-specific antibodies. Cells were stained in 0.5% FBS, HBSS solution with anti-human CD14-BUV737 (clone MΦP9, BD Bioscience (OptiBuild), San Jose, CA, USA) and anti-human CD16-PE-Cy7 (clone 3G8, BD Bioscence (Pharmigen)). Cells were acquired using BD FACSymphony A5 (BD Bioscience) and analyzed using FlowJo (9.3.2) software (BD Bioscience).

### 4.15. Time-Lapse

For time-lapse analysis, mCherry-MCS were cultured for 72 h and then cocultured with CFSE-labeled monocytes (10,000 cells/spheroid) for 2.5 h. After the addition of monocytes, the plate was placed in an incubator and maintained at 37 °C in a humidified 5% CO_2_ atmosphere. Migration of monocytes toward the MCS was monitored every 5 min by Leica THUNDER Imager 3D Live Cell microscope, Wetzlar, Germany. Objective 5×. 

### 4.16. Confocal Microscopy Analysis 

Confocal acquisitions were performed on mCherry-MCS cocultured with or without CSFE-labeled monocytes. MCS were fixed with 4% paraformaldehyde (PFA), at room temperature (RT) for 1 h and mounted with Fluoromount^™^ Aqueous Mounting Medium (Sigma-Aldrich).

MCSs were acquired as z-stack of the entire sections using a Leica TCS SP8 laser scanning confocal microscope, equipped with an HC PL APO CS2 10×/0.40 dry objective. Emission filter bandwidths and sequential scanning acquisition were set up in order to avoid any possible spectral overlap between fluorophores. ImageJ-win64 software package and Imaris (Bitplane, Oxford Instrument Group, Oxfordshire, UK) software were used for image analysis and 3D reconstruction.

### 4.17. Proliferation Assay

MSTO-211H cells were stained with CellTrace™ CFSE Cell Proliferation Kit (Invitrogen Life Technologies Corporation, Eugene, OR, USA) according to the manufacturer’s instructions. Next CFSE-labeled MSTO-211H were seeded in low adherence 96 well to generate MCSs and treated with EPZ (10 μM) or DMSO for 96 h. During the last 48 h, some MCSs were cocultured with monocytes (50,000 cells/spheroids). Next, MCS were then fixed with 4% PFA, RT, for 1 h and mounted with Fluoromount^™^ Aqueous Mounting Medium (Sigma-Aldrich). Confocal acquisitions were performed using Leica SP8 LIGHTNING Confocal Microscope, Wetzlar, Germany. Cell proliferation was quantified in terms of mean of fluorescence (MFI), using ImageJ-win64 software.

### 4.18. Statistical Analysis

Statistical significance between two groups was assessed by unpaired, Student’s *t*-test and among multiple groups by one-way or two-way ANOVA (GraphPad Prism software, San Diego, CA, USA). All statistical assessments were two-sided, and a value of *p*< 0.05 was considered statistically significant.

## Data Availability

The data presented in this study are included in this published article and its additional files. All the data can be shared upon request by email.

## Supplementary Materials

Supplementary materials can be found at https://www.mdpi.com/article/10.3390/ijms22094391/s1.
