# Supplementary material for: Inhibition of the Histone Methyltransferase EZH2 Enhances Protumor Monocyte Recruitment in Human Mesothelioma Spheroids
Source: Int J Mol Sci. 2021 Apr 22;22(9):4391. doi: 10.3390/ijms22094391 (PMC8122808; doi:10.3390/ijms22094391)
Supplement: Supplementary file 1 [file ijms-22-04391-s001.zip › ijms-1179351-supplementary-final.pdf]

**Supplementary Materials**

*In vitro generation of Monocytes-Derived Macrophages (M-DM)*

$CD14^+$ monocytes, were seeded in complete RPMI at the concentration of 2 million cells/ml in low adherence plates and cultured in the presence of human M-CSF (40 ng/mL, Miltenyi) for 6 days. Macrophage differentiation was evaluated by flow-cytometry. $5x10^5$ cells were stained in 0.5% FBS, HBSS solution with: anti-human CD14-FITC (clone M5E2, Bio-Legend) and anti-human CD16-PE (clone 3G8, Bio-Legend) and analyzed by a S3 Cell Sorter (Biorad). When more than 90% CD14+ cells co-express the macrophage differentiation marker CD16, M-DMs were used as following described.

*M-DM gene expression analysis*

To evaluate the effect of EZH2 inhibition on H3K27me3 M-DMs were treated with EPZ-6438 (10 μM, Selleckchem) for the indicated time, then core histone proteins were extracted and analyzed by western blot. Control cells were treated with the same amount of DMSO (Sigma). Due to the low stability of EPZ-6438, for treatments longer than 24 hours, cells were rechallenged with the same dose of drug or vehicle (DMSO) each 24 hours.

To evaluate the effect of EPZ-6438 on M1-polarized activation M-DMs were treated with EPZ-6438 (10 μM) for 24 hours and stimulated with LPS (100ng/ml, Enzo Life Science) during the last 4 hours. Control cells were kept in culture for the entire experimental period. In addition, cells were treated with EPZ (10μM) for 24 hours or maintained untreated for 20 hours and stimulated with LPS (100ng/ml) for the last 4 hours.

To evaluate the effect of EPZ-6438 on M2-polarized activation M-DMs were pre-treated with EPZ-6438 (10μM) for 24 hours and rechallenged with EPZ-6438 along with IL-4 (20ng/ml, Miltenyi) or IL-10 (20ng/ml, Miltenyi) for the following 18 hours. Control cells were kept in culture for the entire experimental period. Additionally, cells were treated with EPZ-6438 (10μM) for 42 hours, and cells were maintained untreated for 24 hours and stimulated with IL-4 or IL-10 (20ng/ml) for the last 18 hours.

*MSTO-211H gene expression analysis*

To evaluate the effect of Mo-TAMs on EMT gene expression of MSTO-211H cells, MCS were pre-treated for 48 hours with EPZ-6438 (10μM) or DMSO then, 50.000 monocytes were added to each MCS and co-cultured for additional 48 hours in presence or absence of EPZ-6438. MCS were mechanically disaggregated and tumor cells were isolated by using Miltenyi Biotech CD14 MicroBeads kit, according to the manufacturer's instructions. MSTO-211H cells, that represented the negative fraction, were analyzed for gene expression by RT-PCR.

**Supplementary Table 1.** Real-Time PCR primers list

| Gene | Forward | Reverse |
|---|---|---|
| ACTB | cccaaggccaaccgcgagaagat | gtcccggccagccaggtccag |
| ARG1 | ctgactggagacctcaagtgc | tcgtggctgtccctttgagaa |
| BAD | cggaggatgagtgacgagt | ccaggactggaagactcgc |
| BAK | aaaatggcttcggggcaag | aacgtagctgcggaaaacct |
| BIM | agtgggtatttctcttttga | gtgtccaattacgccgcaact |
| CCL2 | aagatctcagtgcagaggctcg | cacagatctccttggccacaa |
| CCL5 | gtctttgtcacccgaaag | gacaagagcaagcagaaac |
| CCR2B | gctggtcctgccgctg | cacacgaagcagggttttca |
| CCR5 | ggagccctgccaaaaaatc | tgagtagagcggaggcagga |
| CD163 | agacaaggagctgaggctagt | acagagaccgcttccatgct |
| CD206 | cgatccgacccttccttgac | tgtctccgcttcatgccatt |
| CD47 | tggactgagtctctgtattg | gctagagctaagatacctcaaac |
| CD80 | tttcacttttgaccctaagc | cctgaacagaagtgagaaag |
| COX2 | cccttgggtgtcaaaggtaa | gccctcgcttatgatctgtc |
| CSF1 | ttaagaaggcatttctcctg | ccttgtcatgctcttcataatc |
| CXCL9 | tcttgggcatcatcacttgct | ggtggatagtcccttggttgg |
| CXCL10 | ggaagcactgcatcgattttg | cagaatcgaaggccatcaaga |
| CXCL11 | gccttggctgtgatattgtgt | cactttcactgcttttacccca |
| CXCL12 | acactccaaactgtgcccttc | ccacgtctttgccctttcatc |
| CSFR1 | tgagcaagacctggacaagga | ggatgcaattcttggaagcg |
| CXCR4 | aacttcagtttgttggctg | gtgtatatactgatcccctcc |
| E-CADHERIN | cccttcacagcagaactaac | Cacctctaaggccatctttg |
| HLA-A | aaaaggagggagttacactcagag | gctgtgagggacacatcagag |
| HLA-B | ctaccctgcggagatca | acagccaggccagcaaca |
| IDO | gatgtccgtaaggtcttg | cagtttgccaagacacag |
| IL10 | ttaagggttacctgggttgccaagc | tcttggttctcagcttggggcatca |
| IL12B | cggtcatctgccgcaaa | tgcccattcgctccaaga |
| IL1B | cctactcacttaaagcccgcc | ttagaaccaaatgtggccgtg |
| IL6 | agaacagatttgagagtagtgaggaac | ggcatttgtggttgggtcagg |
| LILRB1 | gcttatgcttatgacctcgaac | agaacaaatctgttgtagcc |
| MMP2 | cacagtccgccaaatgaa | cactcctgagatctgcaaac |
| MMP9 | ccattcacgtcgtccttatg | cgctgggcttagatcattc |
| N-CADHERIN | gatgaaacgccgggataaa | cttcttctcctccaccttct |

| | | |
|---|---|---|
| NOS2 | aaagaccaggctgtcgttga | acgggaccggtatttcattct |
| NOXA | gagatgcctgggaagaagg | ttctccggaagttcagttt |
| P21 | cagcatgacagatttctacc | cagggtatgtatgtacatgagga |
| PD1 | cacgagggacaataggag | atagtccacagagaacacag |
| PDCD1 | caggagggacaataggag | atagtccacagagaacacag |
| PD-L1 | ccgaagtcatctggacaagca | tctcttggaattggtggtggt |
| PUMA | gacgacctcaacgcacagta | cacctaattgggctccatct |
| SIRPA | gaacggaacatctatattgtgg | catgcaaccttgtagaagaag |
| SNAIL | gatgaggacagtgggaaa | ccaaggaagagactgaagtag |
| TNFA | acgaacatccaaccttccca | cccaattctctttttgagccag |
| VEGF | tacctccaccatgccaagtg | atgattctgccctcctccttc |
| VIMENTIN | ccagctaaccaacgacaaa | tcctcttctctctgaagcatctc |

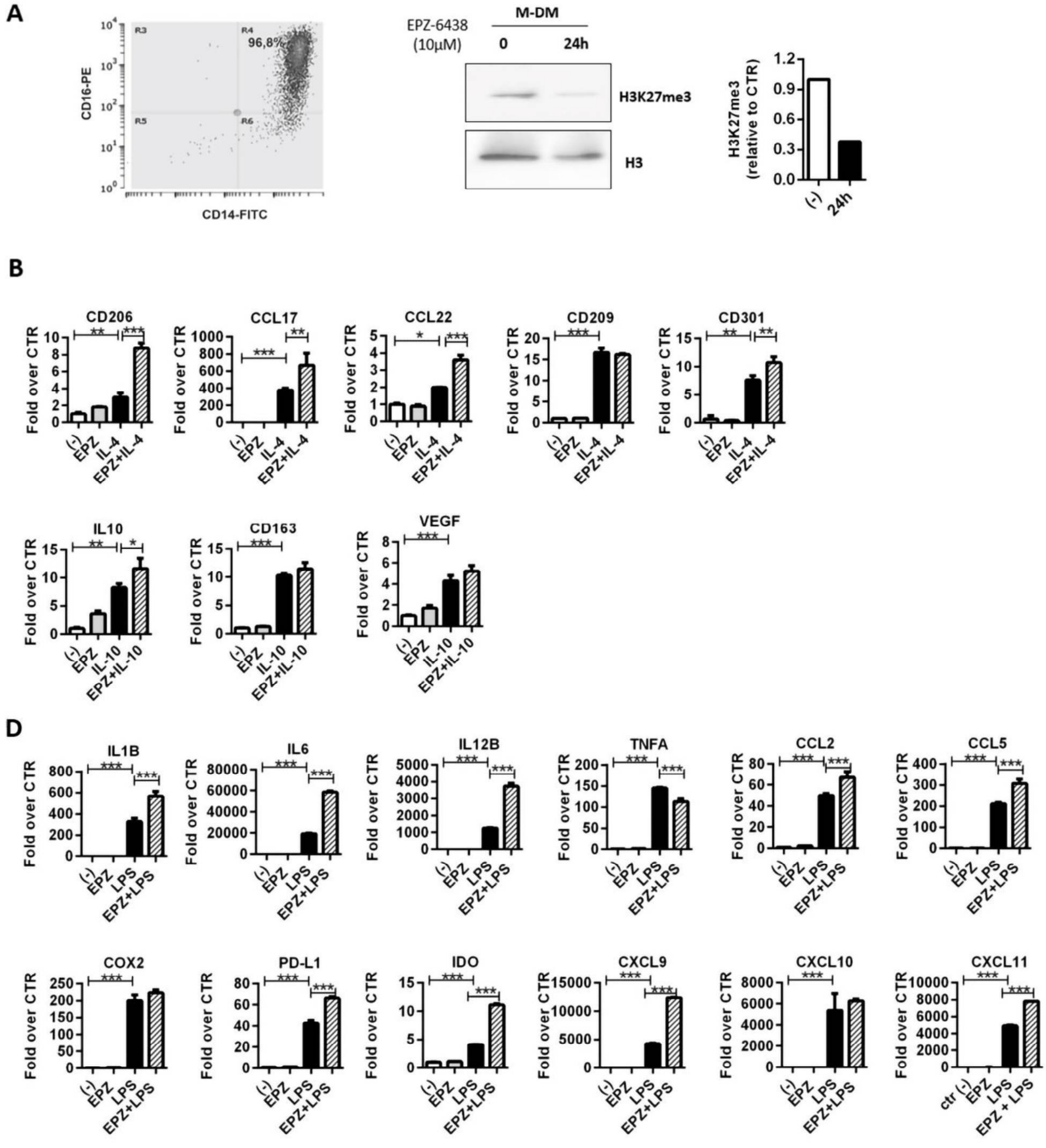


**Figure S1. The effect of EZH2 inhibition affects human M-DM polarized activation.** (**A**) Human monocytes cultured with 40ng/ml of MCSF for 6 days were checked for macrophage differentiation by FACS analysis of CD16 expression. Representative dot plot is shown (A, left). Human M-DM were treated with 10μM EPZ-6438 or DMSO only (-) for 24 hours, then core histone proteins were extracted and analyzed by Western blot for H3K27me3 levels. Histone H3 was used as loading control; (**B, C**) Cells were pre-treated with EPZ-6438 (10μM) for 24 hours and rechallenged with EPZ-6438 along with (**B**) IL-4 (20ng/ml) or (**C**) IL-10 (20ng/ml) for additional 18 hours. Control cells were maintained in standard medium supplemented with DMSO throughout the entire experimental period. An additional group of cells were treated with EPZ-6438 (10μM) for 42 hours and M2-polarized monocytes were cultured for 24 hours and then stimulated with (**B**) IL-4 (20ng/ml) or (**C**) IL-10 (20ng/ml) for the last 18 hours. The expression of selected (**B**) IL-4 (CD206, CCL17, CCL22, CD209 and CD301) and (**C**) IL-10 (IL10, CD163 and VEGF) inducible genes was analyzed by qRT-PCR. β-Actin was used as housekeeping gene. Normalized RT-PCR results are shown as fold increase over control monocytes (-). Data are shown as mean±SD and are representative of one out of four independent experiments with similar results. *$P< 0.05$, **$P < 0.01$ and ***$P< 0.001$ by two-tailed one-way ANOVA, N=3; (**D**) Monocytes were pre-treated with EPZ-6438 (10 μM) for 20 hours,

and then stimulated with LPS (100ng/ml) for the following 4 hours. Control cells were kept in culture in standard medium supplemented with DMSO for the entire experimental period. Additionally, a group of cells was treated with EPZ-6438 (10 μM) for 24 hours and a group of cells was M1-polarized by LPS (100ng/ml) stimulation during the last 4 hours. Monocytes were analyzed to evaluate the expression typical M1-inflammatory genes (TNFA, IL1B, IL6, IL12B, CCL2 and CCL5) by RT-PCR. β-Actin was used as housekeeping gene. Normalized RT-PCR results are shown as fold increase over untreated monocytes (-). Data are shown as mean±SD and are representative of one out of four independent experiments with similar results. * $P < 0.05$, ** $P < 0.01$ and *** $P < 0.001$ by two-tailed one-way ANOVA; N= 3

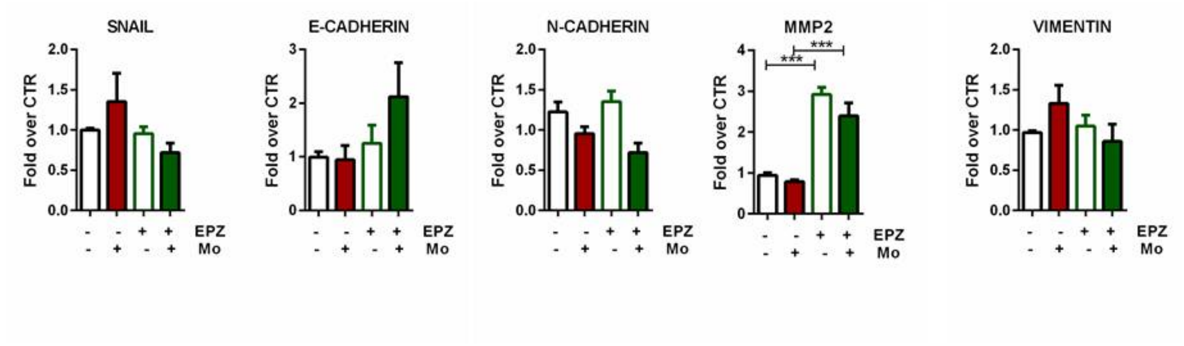


**Figure S2. Mo-TAMs do not alter EMT gene expression by MSTO-211H cells.** RT-PCR analysis of EMT gene expression by MSTO-211H cells derived from MCS co-cultured with monocytes. β-Actin was used as housekeeping gene. Normalized RT-PCR results are shown as fold increase over MSTO-211H cells from DMSO treated MCS (-). Data are shown as mean±SD and are representative of one out of three independent experiments with similar results. *** $P < 0.01$ by two-tailed one-way ANOVA, N= 3.
